# Highly Efficient, Rapid and Co-CRISPR-Independent Genome Editing in *Caenorhabditis elegans*

**DOI:** 10.1534/g3.117.300216

**Published:** 2017-09-11

**Authors:** Harriet Prior, Ali K. Jawad, Lauren MacConnachie, Asim A. Beg

**Affiliations:** * Neuroscience Program, University of Michigan, Ann Arbor, Michigan 48109; † Pharmacology Department, University of Michigan, Ann Arbor, Michigan 48109

**Keywords:** CRISPR, Cas9, ribonucleoprotein, *Caenorhabditis elegans*, disease variants

## Abstract

We describe a rapid and highly efficient method to generate point mutations in *Caenorhabditis elegans* using direct injection of CRISPR-Cas9 ribonucleoproteins. This versatile method does not require sensitized genetic backgrounds or co-CRISPR selection-based methods, and represents a single strategy that can be used for creating genomic point mutations, regardless of location. As proof of principle, we show that knock-in mutants more faithfully report variant-associated phenotypes as compared to transgenic overexpression. Data for nine knock-in mutants across five genes are presented that demonstrate high editing efficiencies (60%), a reduced screening workload (24 F1 progeny), and a rapid timescale (4–5 d). This optimized method simplifies genome engineering and is readily adaptable to other model systems.

Next-generation DNA sequencing technologies have enabled the rapid identification of clinical sequence variants, yet a significant gap still exists in characterizing their functional and pathological significance (Boyd *et al.* 2014). Moreover, allele frequency is often used as a surrogate to infer disease relevance without functional validation in animal models (Minikel and MacArthur 2016). Introducing site-specific variants in a rapid and facile manner in model organisms would greatly aid in unmasking the pathogenic potential of newly identified sequence variants of unknown significance. Recent technological advances such as the CRISPR-Cas9 genome editing system have revolutionized the ability to precisely engineer the genomes of the most prevalent model organisms used in biomedical research (Frokjær-Jensen 2013; Doudna and Charpentier 2014; Sander and Joung 2014; Ma and Liu 2015; Dickinson and Goldstein 2016; Sugi 2016). Here, we have developed a simplified CRISPR-Cas9 genome editing method for generating point mutations in the model organism *Caenorhabditis* *elegans*. This simplified, optimized, and highly-efficient method obviates the need for sensitized genetic backgrounds, selection-based or co-CRISPR methods, and permits the generation of specific knock-in alleles into any strain background within 4–5 d.

Several methods currently exist for engineering the *C. elegans* genome using CRISPR-Cas9, but the majority of these methods rely on specific genetic backgrounds or co-CRISPR strategies in which screening for the successful edit of one marker gene enriches for the genome edit of interest (Dickinson and Goldstein 2016). Although powerful, these methods do have limitations. For example, the *dpy-10* co-CRISPR strategy introduces a point mutation that produces an easily observed dominant roller phenotype (Arribere *et al.* 2014). Although convenient, introducing selectable phenotype-bearing mutations is undesirable if the strain to edit or the desired point mutation of interest itself exhibits a similar phenotype or a phenotype that might be exacerbated or suppressed by the *dpy-10* roller phenotype. Moreover, co-CRISPR strategies rely on creating an additional double-strand break at the marker locus, which not only increases the chance of off-target effects, but may also result in indels within the marker locus (*e.g.*, *dpy-10*) that need to be subsequently repaired. Finally, the highly variable efficiencies (lab-to-lab and day-to-day) present a roadblock for the routine and straightforward generation of knock-in alleles. Thus, we sought to develop a method that precludes co-CRISPR, requires only one targeting single guide RNA (sgRNA), and is completely strain and background independent. The method we present not only reduces the complexity and concentration of CRISPR-Cas9-ribonucleoprotein (RNP) delivery, but also surpasses existing plasmid and RNA-based CRISPR-Cas9 genome editing efficiencies in *C. elegans*. We provide proof of principle that CRISPR-based genomic knock-in more faithfully reports variant associated phenotypes when compared to integration of extrachromosomal arrays—the time-honored gold standard in *C. elegans* (Evans 2006). Last, we present data for nine knock-in alleles across five genes that demonstrate high editing efficiencies (60% average), a significantly reduced screening workload (∼24 F1 progeny), and a rapid timescale (4–5 d) for the generation and detection of the desired knock-in event(s).

## Materials and Methods

### Single guide RNA target selection

sgRNA target sequences (<15 bp from the desired edit) were selected using two freely available CRISPR design prediction programs: (1) http://crispor.tefor.net (Haeussler *et al.* 2016), and (2) the sgRNA analysis tool built into the program ApE (http://biologylabs.utah.edu/jorgensen/wayned/ape/), which utilizes the algorithm by Doench *et al.* (2014). The common top-scoring target sequence shared between these two programs was chosen as the sgRNA for all knock-in alleles generated in this study. An sgRNA for each knock-in allele was ordered (Synthego, Inc.) and resuspended to 50 μM in TE buffer. Supplemental Material, Table S2 lists all sgRNA targeting sequences used in this study.

### Single-stranded oligonucleotide design

Single-stranded oligonucleotides (ssODN) with 40–50 bp 5′ and 3′ homology arms flanking the 20-nucleotide target site were designed containing the desired edit of interest, a unique in-frame restriction site, and conservative nucleotide changes to prevent sgRNA:Cas9 cleavage, and were ordered as Non-PAGE Purified Ultramers from Integrated DNA Technologies (IDT). When possible, the NGG PAM motif was conservatively changed to prevent sgRNA:Cas9 cleavage. All ssODNs were ordered with standard desalting and reconstituted to 100 μM in nuclease-free water. Table S3 lists all ssODN sequences that were used in this study.

### Cas9 protein

Commercially available recombinant *Streptococcus pyogenes* Cas9 nuclease was purchased (IDT) and stored at −20°.

### CRISPR-Cas9-RNP mix and injection

Knock-in alleles were generated using a CRISPR-Cas9-RNP injection mixture containing the following final concentrations: sgRNA (5 μM), Cas9 nuclease (5 μM), ssODN repair template (50 ng/μl), pCFJ90 (2.5 ng/μl), KCl (300 mM), and HEPES (20 mM). These components were homogenously mixed by gentle pipetting and allowed to incubate at room temperature for 10 min. After incubation, the mixture was loaded into a microinjection pipette, and 1-d adult animals were injected in the gonads using the standard microinjection technique (Evans 2006). Injected animals (P0) were allowed to recover for 1–2 hr and were individually transferred to 35 mm nematode growth media (NGM) dishes.

### PCR and restriction enzyme genotyping

Red fluorescent F1 progeny (*n* = 24 total), from successfully injected P0s, were singled to 35 mM NGM dishes and allowed to lay F2 eggs for 2 d. After egg-laying, individual F1s were placed in 7 μl of PCR lysis buffer (50 mM KCl, 10 mM Tris-HCl pH = 8.3, 2.5 mM MgCl_2_, 0.45% NP-40, 0.45% Tween-20) containing 1 mg/ml proteinase K in PCR strip tubes. After 1 hr at −80°, the PCR tubes were lysed for 1 hr at 60°, followed by 15 min at 95° to inactivate the proteinase K. An aliquot (4 μl) of the genomic DNA containing single worm lysate was used to PCR amplify an 820 bp fragment surrounding the targeted genomic edit using Q5 Hot Start High-Fidelity 2X Master Mix (NEB). The primer sequences for *lgc-35* were: F1: 5′-TCGTCATTACGTCCTGGGTTTC-3′ and R1: 5′-CCATTGGTTCAAGACGGGTAAG-3′. PCR products were purified and eluted in 10 μl nuclease-free water using a DNA Clean and Concentrate kit (Zymo Research). The purified and concentrated PCR products were digested with ScaI-HF (NEB) for 1–2 hr and then loaded and separated on a 1.5% agarose gel.

### Molecular biology

The transient fluorescent marker plasmid pCFJ90 (a gift from C. Frojkær-Jensen) contains the *C. elegans myo-2* promoter, a worm-optimized mCherry fluorophore, and an *unc-54* transcriptional terminator sequence that is specifically expressed in pharyngeal muscle. The *lgc-35* genomic rescuing plasmid contains a 3.1 kb upstream promoter sequence, the entire 4.5 kb genomic open reading frame, and a 1.1 kb 3′ untranslated sequence (pAB12) (Jobson *et al.* 2015). The Q5 site-directed mutagenesis kit (NEB) was used to engineer the L324S mutation in the pAB12 genomic rescue plasmid. Colonies were subsequently prepared and Sanger sequenced to identify a correctly edited and error-free plasmid containing the L324S mutation (pJC08).

### Extrachromosomal transgene expression and UV integration

The plasmids pCFJ90, pAB12, and pJC08 were purified using the Zyppy Plasmid Miniprep Kit (Zymo Research), and eluted in nuclease-free water. L324S extrachromosomal array-expressing animals were generated by injecting 1-d adult animals in the gonads with a mixture containing a final concentration of: pCFJ90 (2.5 ng/μl) + pJC08 (30 ng/μl) + 1 kb+ DNA Ladder (67.5 ng/μl; Life Technologies). Independent extrachromosomal transgenic lines were generated by injecting the DNA mix into *lgc-35* (*tm1444*) deletion animals. To integrate the transgene, extrachromosomal expressing lines were UV-irradiated (Stratagene) as previously described (Evans 2006). Three integrated lines were isolated and outcrossed 6–10 times to the N2 wild-type strain.

### Electrophysiology

The *C. elegans lgc-35* cDNA was isolated and cloned into the *Xenopus laevis* expression vector pSGEM (a gift from M. Hollman) as previously described (Jobson *et al.* 2015; Nicholl *et al.* 2017). All animal care and experimental procedures followed the guidelines from the National Institutes of Health (NIH) and the Institutional Animal Care and Use Committee (IACUC) at the University of Michigan. Defolliculated stage V–VI *X. laevis* oocytes were purchased from *Xenopus1* (Dexter, MI), and injected with 12.5 ng of LGC-35 (wild type or L324S) capped RNA (cRNA). Injected oocytes were incubated for 2–5 d at 18° in Barth’s solution: 88 mM NaCl, 1 mM KCl, 0.33 mM Ca(NO_3_)_2_, 0.41 mM CaCl_2_, 0.82 MgSO_4_, 2.4 mM NaHCO_3_, 10 mM HEPES, supplemented with 1 mM Na^+^-pyruvate, and 50 mg/liter gentamicin (pH = 7.4, NaOH). The standard bath solution for dose-response experiments was Frog Ringer’s: 115 mM NaCl, 2 mM KCl, 1.8 mM CaCl_2_, 10 mM HEPES (pH = 7.4, NaOH). Standard two-electrode voltage clamp recordings were performed as previously described (Nicholl *et al.* 2017).

### Sequence alignments

Protein sequences were aligned using the ClustalW algorithm in the MacVector software suite. The GenBank identifier (GI) numbers for the protein sequences used for comparative sequence analysis were: LGC-35 (71998246), GABRA1 (27808653), GABRB2 (292495010), and GABRG2 (189083762).

### Worm strains

For all experiments, the Bristol N2 strain was used as the wild-type control and was the parental strain for all transgenic lines generated in this study. Worms were grown at 20° on NGM plates seeded with the OP50 *Escherichia coli* strain. The Bristol N2 strain was obtained from the *Caenorhabditis* Genetics Center (CGC), which is funded by the NIH Office of Research Infrastructure Programs (P40 0D010440).

### Statistical analysis

Statistical analyses were performed using GraphPad Prism 7. One-way ANOVA with Tukey *post hoc* test was used for comparison involving >2 groups. Data are reported as mean ± SEM, and all experiments were performed blind to the observer.

### Data availability

Strains generated in this study are available on request. The authors state that all data necessary for confirming the conclusions presented in article are fully represented within the article and the Supplemental Material.

## Results

**Figure 1:**
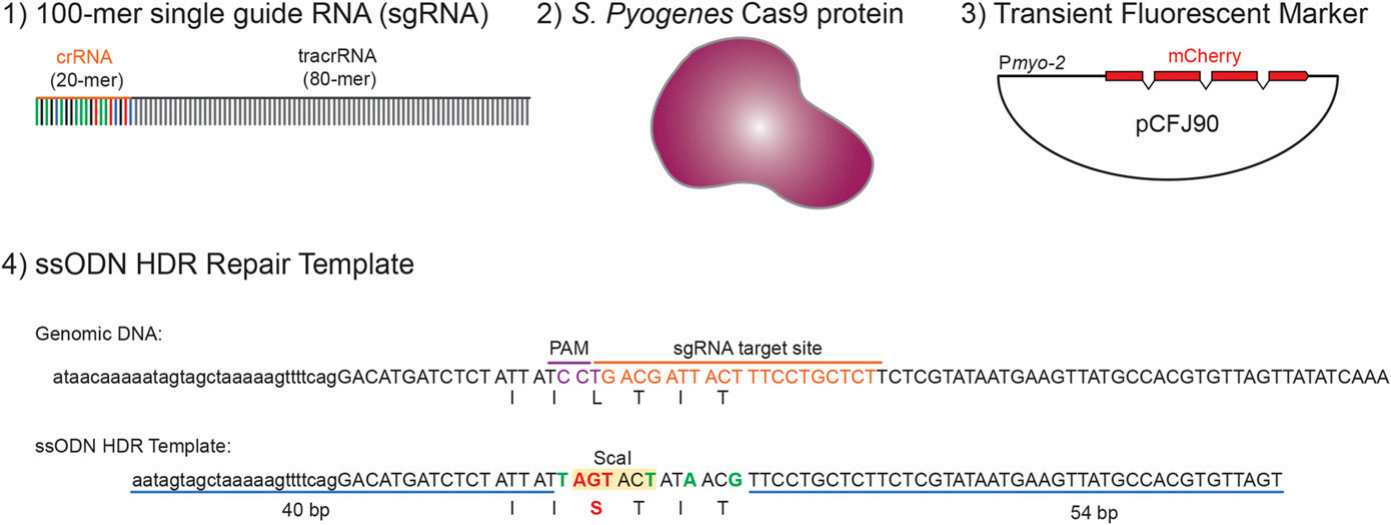
CRISPR-Cas9-RNP injection components. Schematic illustration of the four-component RNP injection mix: (1) 100-mer sgRNA, which is a synthetic oligonucleotide containing both the crRNA (colored) and tracrRNA (black) sequences; (2) *S. pyogenes* Cas9 protein; (3) a transient fluorescent plasmid (pCFJ90) that enriches for F1s that have taken up the RNP payload, and (4) an ssODN HDR template that contains the desired edit (red), conservative changes to the PAM motif (purple), and conservative changes (green) that introduce a unique ScaI restriction enzyme site (yellow box) and prevent cleavage by the sgRNA:Cas9 complex. The sense genomic DNA strand is shown as a reference, and the PAM (purple) and sgRNA target sites (orange) are highlighted.

**Figure 2:**
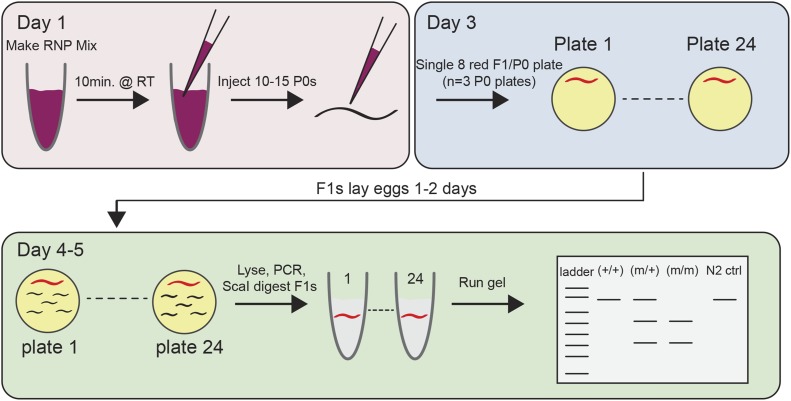
CRISPR-Cas9-RNP workflow. Schematic illustration of the 4–5 d workflow for creating CRISPR-Cas9-RNP knock-in alleles.

The direct delivery of precomplexed CRISPR-Cas9 RNPs has several distinct advantages over plasmid, RNA, or viral-based delivery methods; most notably, bypassing the need for cellular transcription and translation. Accordingly, we optimized a method that consists of only four components: (1) a single-stranded 100-mer sgRNA oligonucleotide, (2) the *S. pyogenes* Cas9 protein, (3) an inert and transient fluorescent marker, and (4) an ssODN homology-directed repair (HDR) template (Figure 1). First, we simplified the delivery of the targeting sgRNA, which normally consists of two components; a CRISPR RNA (crRNA) that is complementary to the target sequence, and a transactivating CRISPR RNA (tracrRNA), which duplexes with and enables maturation of the crRNA (Doudna and Charpentier 2014). The crRNA and tracrRNA molecules must anneal to form a functional sgRNA that directs the Cas9 nuclease to the targeted protospacer adjacent motif (PAM) for site-specific chromosomal cleavage. Annealing efficiency of this bipartite RNA system (crRNA:tracrRNA) is never 100% efficient, which is further complicated as *in vitro* transcribed crRNAs can dimerize (Dang *et al.* 2015) and unannealed tracrRNA fragments can form tetrameric complexes (K. Holden, personal communication). Together, these impediments may negatively interfere with Cas9 protein activity and decrease editing efficiencies. Thus, we used synthetic sgRNA molecules that contain both the crRNA and tracrRNA sequences as a single 100-mer oligonucleotide that requires no annealing (Figure 1). Second, we increased the ionic strength of the injection mix (300 mM KCl) to prevent Cas9 protein aggregation (Anders *et al.* 2014; Paix *et al.* 2017), which was a major impediment to repeatable and successful injection due to clogging of the micropipette tip. Third, we reduced the molar concentration of sgRNA:Cas9 RNP delivery to decrease the likelihood of off-target effects, while still maintaining high editing efficiencies. The majority of RNP-based delivery methods in *C. elegans* use high concentrations of Cas9 nuclease (15–25 μM) and targeting sgRNAs (30–40 μM) (Cho *et al.* 2013; Paix *et al.* 2015, 2017). We reduced the final sgRNA and Cas9 nuclease concentrations to 5 μM while retaining high editing efficiencies. Fourth, we included a transient fluorescent plasmid (P*myo-2*::mCherry) as a fiducial marker of successful RNP payload uptake (Figure 1). The fluorescent marker enriches for F1 progeny that have the highest likelihood of containing the CRISPR edit, and this marker is lost in the subsequent generation. Fifth, we designed ssODN repair templates containing both the desired edit of interest and a unique in-frame restriction enzyme site to easily identify modified F1 progeny by PCR restriction digest analysis (Figure 1). These components are simply mixed together, incubated at room temperature for 10 min, and then injected into the gonad of *C. elegans* using the standard microinjection technique (Mello *et al.* 1991) (Figure 2 and File S1).

**Figure 3:**
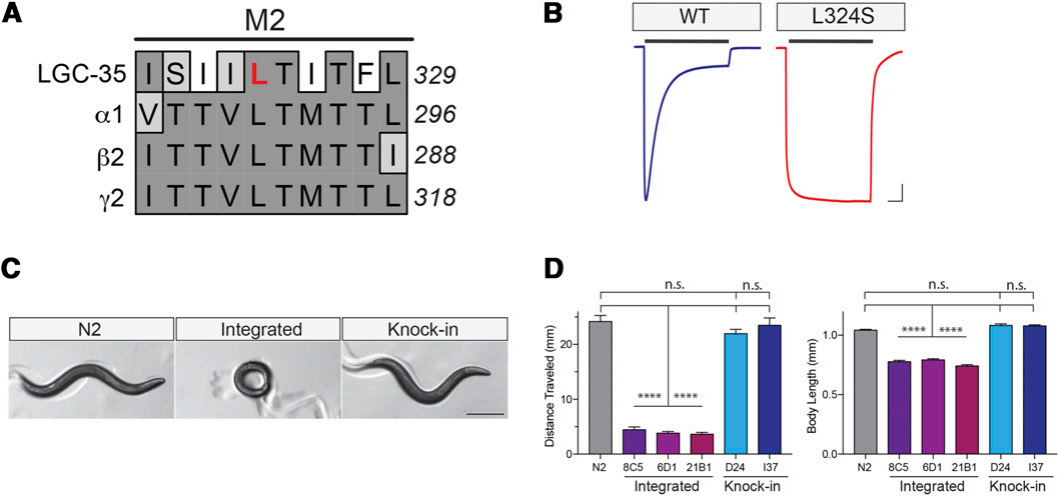
*lgc-35*(L324S) homozygous knock-in animals exhibit less severe phenotypes than extrachromosomal or integrated transgene overexpression. (A) Sequence alignment of LGC-35 with human GABA receptor subunits. The conserved leucine 324 in LGC-35 transmembrane 2 (M2) is highlighted in red. (B) Representative traces from *X. laevis* oocytes expressing either LGC-35 (wild type) or LGC-35 (L324S). Black bar denotes a 6-min application of saturating concentration of 1 mM GABA. The L324S mutation causes a complete block of receptor desensitization compared with wild-type controls. Bar, 1 μA, 1 min. (C) Representative images of wild-type (N2), L324S integrant (8C5), and L324S homozygous CRISPR knock-in animals. Note the small body size and coiled phenotype present in the L324S integrant line compared with both N2 and L324S homozygous knock-in lines. Bar, 250 μm. (D) Left, total distance (millimeter) moved over a 2-min period was quantitated. Three independent L324S UV-integrated lines (8C5, 6D1, 21B1) were compared against wild type (N2) and two independent L324S CRISPR knock-in lines (D24, I37). Right, body size was quantitated, revealing that integrated lines are smaller than N2 or CRISPR knock-ins. One-way ANOVA, Tukey *post hoc*, **** *P* < 0.0001, n.s., not significant, error bars are SEM. At least *n* ≥ 40 animals per line/genotype were quantitated.

As proof of principle, we targeted the *lgc-35* gene, which encodes an excitatory cation-selective homopentameric ionotropic GABA-gated ion channel that modulates the worm locomotor circuit (Jobson *et al.* 2015). We identified that a Leu324Ser (L324S) mutation within the pore-forming domain of LGC-35 completely blocked receptor desensitization when expressed in *X. laevis* oocytes (Figure 3, A and B). We reasoned that prolonged channel opening in an LGC-35(L324S) mutant might produce a locomotion phenotype due to sustained motor neuron depolarization. To compare our CRISPR-based method against conventional exogenous transgene expression, we first overexpressed an *lgc-35* (L324S) point-mutated genomic fragment in an *lgc-35*(*tm1444*) null background using standard extrachromosomal transgene expression. Previously, we demonstrated that the parental wild-type genomic construct rescues *lgc-35* mutant loss-of-function phenotypes when expressed in null mutants, demonstrating that transgenic reexpression of the receptor is functional and expressed in the correct cell types (Jobson *et al.* 2015). Several *lgc-35*(L324S) extrachromosomal expressing lines were established and all displayed a striking locomotor phenotype. To ensure these phenotypes were not due to differential expression of the extrachromosomal transgene, we created several independent integrated lines that phenocopied extrachromosomal transgene overexpression phenotypes: severely reduced locomotion, small body size, delayed growth, and profound body coiling (Figure 3, C and D).

**Figure 4:**
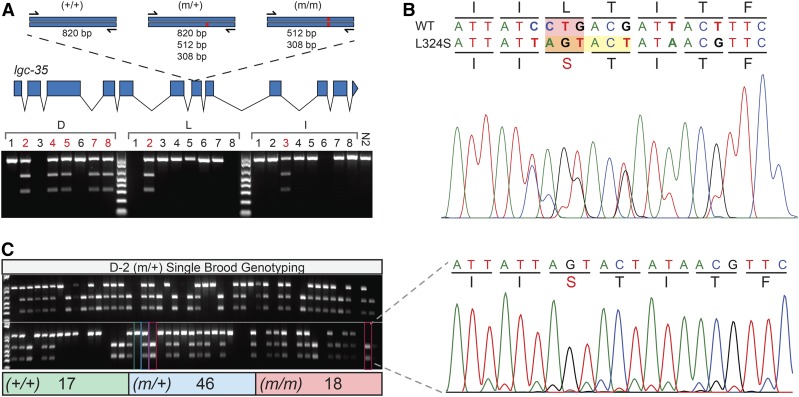
*lgc-35* (L324S) CRISPR-Cas9-RNP genotyping and Mendelian segregation. (A) Top, exon-intron structure of the *lgc-35* genomic locus and the PCR strategy used for identifying edited animals. PCR primers were designed to produce a single 820 bp product that flanks the edit of interest. Wild-type alleles (+/+) lacking the edited and ScaI restriction site show a single 820 bp band. Successfully edited heterozygotes (m/+) produce three bands (820, 512, and 308 bp), and homozygotes (m/m) produce two bands (512 and 308 bp) when digested with ScaI. Bottom, genotyping data from the 24 positive red fluorescent progeny chosen from three different P0 injected animals (D, L, I). PCR restriction digest analyses revealed that F1 animals D-2,4,5,7,8, L-2, and I-3 are heterozygous knock-ins. An N2 control is shown at the far right of the gel. PCR failures occurred for animals D-3, L-1,8, and I-6. (B) Representative chromatogram from heterozygous animal D-2. Nucleotide codon spacing and amino acid translations are shown above for the heterozygote and wild-type strands. The desired edit is highlighted by a red box, and the conservative changes that create an in-frame ScaI restriction site are highlighted by a yellow box. (C) Left, Mendelian analysis from animal D-2. 96 single F2 progeny were picked and genotyped. The total for the analysis was 81, since there were 15 PCR failures. Mendelian segregation reveals 17 wild-type (+/+), 46 heterozygote (m/+), and 18 homozygote (m/m) animals. The green outline box shows representative wild type (+/+), the blue outline box shows representative heterozygotes (m/+), and red outline box shows representative homozygotes (m/m). Right, representative chromatogram from a homozygous knock-in animal.

Having established that overexpression of LGC-35 (L324S) produced a severe locomotor phenotype, we next engineered this mutation into the endogenous gene (Figure 1 and Figure 4A). We injected 15 P0 animals, and selected three plates that exhibited a high frequency of fluorescent F1 progeny (Figure 2). Eight fluorescent F1 progeny were singled per P0 plate for a total of 24 F1 positives. After 2 d of egg-laying, each F1 was lysed to isolate genomic DNA and subjected to PCR with primers flanking the targeted site (Figure 2 and Figure 4A). The ssODN HDR template was designed to include both the desired mutation and a unique in-frame ScaI restriction site (Figure 1 and Figure 4B). In correctly edited animals, ScaI digestion cuts the full-length PCR product (820 bp) into two bands of 512 bp and 308 bp (Figure 4A). This genotyping strategy unambiguously differentiates between wild-type (802 bp), heterozygote (802, 512, and 308 bp) and homozygote (512 and 308 bp) animals (Figure 2 and Figure 4A). PCR and subsequent ScaI restriction analyses of the 24 F1s revealed seven putative heterozygous animals (29%) (Figure 4A). Sanger sequencing revealed that each heterozygote contained the precise genome edit that was designed in the ssODN repair template (Figure 4B). Next, we singled 96 F2 progeny from a single heterozygote and performed PCR and restriction analyses, revealing Mendelian segregation and recovery of homozygous knock-in animals (Figure 4C). Having confirmed accurate editing and heritable transmission, we examined the behavioral phenotype of homozygous *lgc-35*(L324S) knock-in animals. Superficially, the homozygous knock-in mutants did not exhibit a phenotype that was comparable to the striking phenotypes observed in the integrated overexpressing strains. To quantitatively compare the impact of the L324S mutation in the context of endogenous genomic expression *vs.* transgene overexpression, we measured total distance moved over time as a function of motility and body size (Figure 3D). Intriguingly, the knock-in strains were similar to wild-type controls in both total distance moved and body size (Figure 3D).

Using the same strategy and workflow, we validated the fidelity and ease of our method by generating eight additional knock-in variants (human and experimental) across four separate genes (Table S1). Knock-in mutations were confirmed by PCR genotyping, restriction digest analysis, and Sanger sequencing of the entire genomic open reading frame using the workflow shown in Figure 2. Significantly, the average success rate from these experiments was 60% (Table S1), further demonstrating the efficiency of our method. Remarkably, we observed many biallelic edits in positive F1 progeny, which corroborates the high efficacy of the method (data not shown). Thus, our strategy is not limited to particular loci, as we achieved highly efficient and precise genome editing across several genes dispersed throughout the genome.

## Discussion

Here, we developed and optimized a streamlined method to rapidly and efficiently generate point mutations in *C. elegans* using CRISPR-Cas9-RNP delivery. We demonstrate that our method exhibits no bias in editing genomic loci, significantly reduces screening workload, reduces the concentration of both sgRNA and Cas9 nuclease and is completely strain independent. This simple method does not require specialized equipment, making it possible to standardize the generation of CRISPR-Cas9-based point mutants, which should increase the reproducibility and cross-validation within and across laboratories. Importantly, genomic studies are rapidly uncovering variants of unknown significance that may be associated with disease; however, functional validation of these variants has sorely lagged. Our method presents an inroad to bridging this gap, and feasibly permits the production and testing of variants of unknown significance to scale in *C. elegans* models. Finally, we provide data demonstrating that knock-in alleles more faithfully represent variant-associated phenotypes compared with transgenic overexpression.

These data clearly demonstrate that *lgc-35* (L324S) integrated and extrachromosomal transgene phenotypes differ significantly from homozygous knock-ins. One possible explanation is that transgene overexpression, either through extrachromosomal delivery or multi-copy integration, drives nonphysiological receptor levels that cause toxicity or mitigate homeostatic compensation. Additionally, high transgene expression can result in both ectopic cellular expression and inappropriate subcellular localization, which may account for the more severe behavioral phenotypes observed in the L324S-overexpressing transgenics, as compared with the homozygous knock-in strains. Together, these proof-of-principle data demonstrate that knock-in alleles more faithfully report variant-associated phenotypes in comparison with either extrachromosomal or integrated transgene overexpression. Notably, we did not encounter any bioinformatically chosen sgRNA targeting sequence that failed, which offers the distinct feasibility to scale clinical and experimental variant knock-in production. In conclusion, our simplified genome editing method dispenses with the need for specialized strains, obviates coconversion strategies that rely on creating an additional double-strand break, significantly reduces RNP concentrations, and increases workflow efficiency and efficacy for creating knock-in mutations. The tools, methods, and workflow described here are readily adaptable to other nematode species and likely amenable to other experimental model systems in which CRISPR-Cas9 editing is utilized.

## Supplementary Material

Supplemental material is available online at www.g3journal.org/lookup/suppl/doi:10.1534/g3.117.300216/-/DC1.
