# Supplementary material for: Highly Efficient, Rapid and Co-CRISPR-Independent Genome Editing in Caenorhabditis elegans
Source: G3 (Bethesda). 2017 Sep 11;7(11):3693–8. doi: 10.1534/g3.117.300216 (PMC5677160; doi:10.1534/g3.117.300216)
Supplement: Supplementary file 1 [file 3693TableS1.docx]

| gene (edit) | Screened P0 Plate | Positive F1s | Edited Event | % Success |
| --- | --- | --- | --- | --- |
| *lgc-35* (L324S) | 3 | 24 | 7 | 29 |
| *aars-2* (G935S) | 3 | 20 | 19 | 95 |
| *aars-2* (G102R) | 6 | 18 | 11 | 61 |
| *sod-1* (N66S) | 3 | 36 | 24 | 67 |
| *sod-1* (A96T) | 4 | 36 | 18 | 50 |
| *sod-1* (D84V) | 3 | 17 | 9 | 53 |
| *tdp-1* (R219A) | 1 | 1 | 1 | 100 |
| *ric-8* (S435A) | 1 | 6 | 5 | 83 |
| *ric-8* (S440A) | 5 | 18 | 11 | 61 |
| **Average** |  | 176 | 105 | **59.6** |

Supplementary Table 1. Editing efficiencies using the CRISPR-Cas9-RNP method
