# Supplementary material for: Highly Efficient, Rapid and Co-CRISPR-Independent Genome Editing in Caenorhabditis elegans
Source: G3 (Bethesda). 2017 Sep 11;7(11):3693–8. doi: 10.1534/g3.117.300216 (PMC5677160; doi:10.1534/g3.117.300216)
Supplement: Supplementary file 2 [file 3693TableS2.docx]

Supplementary Table 2. sgRNA target sequences

| Gene (edit) | sgRNA target sequence |
| --- | --- |
| *lgc-35* (L324S) | AGAGCAGGAAAGTAATCGTC |
| *aars-2* (G935S) | GTTCTCGGTGGAAAAGGTGG |
| *aars-2* (G102R) | CACACATACTTCGAGATGCT |
| *sod-1* (N66S) | GGTCCACACTTCAATCCATT |
| *sod-1* (A96T) | CTTGATTTTTGCCACTCCAT |
| *sod-1* (D84V) | TTCCTAGATCGCCTACGTGA |
| *tdp-1* (R219A) | TTTGTCAGAATGTCATCAGT |
| *ric-8* (S435A) | AAAACACGCGTCGGACAGTG |
| *ric-8* (S440A) | AAAACACGCGTCGGACAGTG |
