# Supplementary material for: Highly Efficient, Rapid and Co-CRISPR-Independent Genome Editing in Caenorhabditis elegans
Source: G3 (Bethesda). 2017 Sep 11;7(11):3693–8. doi: 10.1534/g3.117.300216 (PMC5677160; doi:10.1534/g3.117.300216)
Supplement: Supplementary file 3 [file 3693TableS3.docx]

Supplementary Table 3. ssODN homology directed repair template sequences

| Gene (edit) | ssODN Sequence |
| --- | --- |
| *lgc-35* (L324S) | gcggaatatcgataacaaaaatagtagctaaaaagttttcagGACATGATaTCTATTATatTGACaATaACgTTCtTttTgTTCagcTAcAAcGAAGTTATGCCACGTGTTAGTTATATCAAAGCGATGGATGTTTATTTAGg |
| *aars-2* (G935S) | GGTGAGCAACGGCCTGAAAGCCAACGAGTGGGTGAATGAAGTTTGCACAGTaCTtGGaGGtAAgaGcGGcGGAAAGGACGCAAATGCTCAGCTCACCGGAGAGAATGTCGATAAACTTGAT |
| *aars-2* (G102R) | CCGGTGGAAAGCATAACGACTTGGACGATGTCGGCAAAGATGTCTACCATCAtACgTACTTCGAaATGtTgcGcAATTGGTCTTTCGGTGATTATTTCAAGgttagaaattgatttagttaattaa |
| *sod-1* (N66S) | aattcaagcgatataaaaaatttccatacTTTTGGTCCACCATGAGTCTTTCCgAAgGGagaGAAaTGgGGcCCGGCAGAAATGCATCCGTTGGTGGAATCACCATATTGGTGAACGTGGAATCC |
| *sod-1* (A96T) | GCCAACGACAGTGTTTGGACCGTAAAGCGTGACGAGCGTGTCGGTGAGCTTaATcTTgGtgACcCCgTCaGCTCCAGCTTCCACATTTCCTAGATCGCCTACGTGACGGATCTCGGActga |
| *sod-1* (D84V) | CGTGTCGGTGAGCTTGATTTTTGCCACTCCATCGGCTCCAGCTTCCACATTTCCaAGtaCtCCgACGTGtCtaATCTCGGActgaaaaaatattataattcaagcgatataaaaaatttccata |
| *tdp-1* (R219A) | tcttcttcagATAAAACGAAAGTCGGATGGAAACTCAAAAGGATTTGGATTcGTtgcgATGagcTCtGTaGGTGAACAAAATAAAGTATTAGCTATCCCACAGCACATGATTGATGGTCG |
| *ric-8* (S435A) | GTCACCTCGCAAATTTGGGTCTTCTCGGACAAATCAATCAACCAAAACACGCagCtGACAGTGAaGATAGTGAAACTGAGGATTATAATCAGATTAAGGATAGgtatgctatactttttgtgc |
| *ric-8* (S440A) | GTCACCTCGCAAATTTGGGTCTTCTCGGACAAATCAATCAACCAAAACACGCtagcGACtcTGAaGATgccGAAACTGAGGATTATAATCAGATTAAGGATAGgtatgctatactttttgtgc |
