# Supplementary material for: Highly Efficient, Rapid and Co-CRISPR-Independent Genome Editing in Caenorhabditis elegans
Source: G3 (Bethesda). 2017 Sep 11;7(11):3693–8. doi: 10.1534/g3.117.300216 (PMC5677160; doi:10.1534/g3.117.300216)
Supplement: Supplementary file 4 [file 3693FileS1.docx]

Supplementary Protocol: CRISPR-Cas9 RNP Injection Protocol

**Reagent Vendor**

Single guide RNA Synthego, Inc.

ssODN HDR Template Integrated DNA Technologies

*S. Pyogenes* 3x-NLS-Cas9 Integrated DNA Technologies

pCFJ90 Plasmid Addgene (#19327)

KCl (4M) Sigma (P5405)

HEPES (0.5M) Sigma (H4034)

Nuclease Free Water Synthego, Inc.

Nuclease Free TE Synthego, Inc.

Zyppy Miniprep DNA kit Zymo Research

DNA Clean and Concentrate kit Zymo Research

Gel extraction kit Zymo Research

Q5 Hot Start 2X High-Fidelity Master Mix New England Biolabs

Worm Lysis Buffer Homemade (see below)

Proteinase K Sigma (P2308)

Worm Lysis Buffer:

| Reagent | [Final] |
| --- | --- |
| KCl | 50 mM |
| Tris-HCl pH=8.3 | 10 mM |
| MgCl_2_ | 2.5 mM |
| NP-40 | 0.45% |
| Tween-20 | 0.45% |
| Proteinase K | 1mg/ml |

- Add Proteinase K fresh before use

**I. Resuspend sgRNA and ssODN**

- Resuspend lyophilized sgRNA to 50μM in Nuclease-free TE
- Resuspend lyophilized ssODN to 100μM in Nuclease-free water

**II. Prepare Injection Mix**

- Centrifuge reagents at max speed for 3 minutes.
- Add reagents in the following order:

| Reagent | Volume | Final Concentration |
| --- | --- | --- |
| H_2_0 | 12.57 μl | -- |
| KCl (4M) | 1.88 μl | 300 mM |
| HEPES (0.5M) | 1 μl | 20 mM |
| pCFJ90 (25ng/μl) | 2.5 μl | 2.5 ng/μl |
| ssODN (500ng/μl) | 2.5 μl | 50 ng/μl |
| sgRNA (50μM) | 2.5 μl | 5 μM |
| Cas9 (61μM) | 2.05 μl | 5 μM |
| Final Volume | 25 μl |  |

- Mix thoroughly until the solution is visibly homogenous
- Incubate for 10 minutes @ room temperature

**III. Injection Protocol**

- The day before injection, pick healthy late L4 animals to an NGM plate.
- Load an injection micropipette with the injection mix.
- Break pipette tip so that ~30 psi gives an even flowing solution. *Note: Large diameter tips adversely affect animal health and decrease F1 progeny yield.
- Inject 10-15 1-day adult animals in both gonads.
- Allow injected P0s to recover for 1-2 hours and then single to 35mm NGM plates seeded with fresh OP50 bacteria

**IV. Screen P0 plates for successful injection**

- ~1-2 days post-injection, screen the P0s plates for the presence of F1 progeny expressing mCherry in the pharyngeal muscles.
- Choose 3 P0 plates that show the highest number of positive F1 progeny.

**V. Single mCherry positive F1s**

- Single 8 red F1 progeny/P0 plate for a total of 24 F1 animals

*Example:* Top three P0 plates = #1, 6, 9

F1 plates = 1-1, 1-2, 1-3, 1-4, 1-5, 1-6, 1-7, 1-8

6-1, 6-2, 6-3, 6-4, 6-5, 6-6, 6-7, 6-8, 6-9

9-1, 9-2, 9-3. 9-4, 9-5, 9-6, 9-7, 9-8, 9-9

- Let F1s lay eggs for 1-2 days

**VI. Lyse, PCR and restriction genotype F1 animals.**

- Pipette 7 μl of Worm Lysis Buffer + Proteinase K into PCR strip tube caps.
- Pick individual F1s from each plate and place into the Worm Lysis buffer using a dissecting stereomicroscope. Attach PCR strip tubes to caps and seal. **Make sure to only transfer the F1,* ***NO*** *eggs or F2 progeny.*
- Spin PCR tubes in a tabletop centrifuge for 1 minute at maximum speed
- Freeze tubes at -80°C for 1 hour. Worms can be stored indefinitely at this point.
- Lyse animals for 60 minutes @ 65°C, followed by 15 minutes @ 95°C
- Set up PCR reactions as follows:

| Reagent | 1X |
| --- | --- |
| 2X Q5 Mastermix | 12.5 μl |
| Primer F1 (10μM) | 1.25 μl |
| Primer R1 (10μM) | 1.25 μl |
| Worm Lysate | 4 μl |
| H_2_0 | 6 μl |
| Final Volume | 25 μl |

- Determine primer annealing temperature and extension time per manufacturer guidelines.
- Purify PCR products using the DNA clean and concentrate kit per the manufacturer protocol.
- Elute purified PCR product in 10μl of nuclease-free water.
- Digest PCR products using restriction enzyme site that was engineered into the ssODN HDR template:

| Reagent | 1X |
| --- | --- |
| 10X Enzyme Buffer | 2 μl |
| DNA | 10 μl |
| H_2_O | 7 μl |
| Restriction Enzyme | 1 μl |
| Final Volume | 20 μl |

- Digest PCR products for 1-2 hours at the appropriate temperature.
- Load and run digested PCR product on a 1% agarose gel to identify potential edited F1 animals.

**VII. Sanger Sequencing Verification**

- Once potential heterozygous or homozygous F1s have been identified, use the remaining F1 worm lysate(~3μl) and replicate the PCR. ** Do not cut the PCR product with restriction enzyme*
- Run the PCR product on a 1% agarose gel, and gel extract the single band.
- Gel purify the PCR product and Sanger sequence with a nested primer that is located inside Primers F1 and R1.
